# Do nursery habitats provide shelter from flow for juvenile fish?

**DOI:** 10.1371/journal.pone.0186889

**Published:** 2018-01-17

**Authors:** Darren M. Parsons, Iain MacDonald, Dane Buckthought, Crispin Middleton

**Affiliations:** 1 NIWA, Auckland, New Zealand; 2 Institute of Marine Science, University of Auckland, Auckland, New Zealand; 3 NIWA, Hamilton, New Zealand; 4 NIWA, Bream Bay, Ruakaka, New Zealand; University of Windsor, CANADA

## Abstract

Juvenile fish nurseries are an essential life stage requirement for the maintenance of many fish populations. With many inshore habitats globally in decline, optimising habitat management by increasing our understanding of the relationship between juvenile fish and nursery habitats may be a prudent approach. Previous research on post–settlement snapper (*Chrysophrys auratus*) has suggested that structure may provide a water flow refuge, allowing snapper to access high water flow sites that will also have a high flux of their pelagic prey. We investigated this hypothesis by describing how Artificial Seagrass Units (ASUs) modified water flow while also using a multi–camera set up to quantify snapper position in relation to this water flow environment. Horizontal water flow was reduced on the down–current side of ASUs, but only at the height of the seagrass canopy. While the highest abundance of snapper did occur down–current of the ASUs, many snapper also occupied other locations or were too high in the water column to receive any refuge from water flow. The proportion of snapper within the water column was potentially driven by strategy to access zooplankton prey, being higher on the up–current side of ASUs and on flood tides. It is possible that post–settlement snapper alternate position to provide opportunities for both feeding and flow refuging. An alternative explanation relates to an observed interaction between post–settlement snapper and a predator, which demonstrated that snapper can utilise habitat structure when threatened. The nature of this relationship, and its overall importance in determining the value of nursery habitats to post–settlement snapper remains an elusive next step.

## Introduction

For many fish species nursery habitats represent a critical life history requirement that contributes to the maintenance of fish populations by supporting juvenile fish through a vulnerable early life–history stage [1–4]. Protection from predation, and an increased availability of food are generally put forward to explain this nursery habitat association [2]. Many of the habitats that serve as nurseries are biogenic in nature, consisting of structure–forming seagrasses, bivalves, or sponges, and are generally located in inshore locations. As such, many of these habitats, and the value they provide to fish populations, are at risk of decline [5–8].

Whilst the maintenance of nursery habitats might best be informed by a complete understanding of the contribution of different locations or types of nursery habitat to adult fish populations [2], this has largely proven to be an elusive goal [9]. Alternatively, understanding more detail around the mechanisms that drive the relationship between juvenile fish and nursery habitat, could represent a prudent way of guiding habitat management to conserve or restore the aspects of habitats that juvenile fish value [10].

Snapper, *Chrysophrys auratus*, (= *Pagrus auratus*) (F. Sparidae), are a recreationally and commercially important fish species that are abundant in the coastal water of northern New Zealand [11]. Post–settlement stage snapper (<60 mm Fork Length (FL)) spend their first few months as benthic juveniles occupying shallow estuarine locations before dispersing to a range of habitats/locations. During this post–settlement stage high abundances are associated with habitat structure, whereas snapper may be almost completely absent at immediately adjacent bare sediment sites that are only tens of metres away [12–14]. The predators of post–settlement snapper are largely unknown, with observations of predation rare [15]. Furthermore, post–settlement snapper predominantly feed on pelagic crustaceans [15, 16] that are not connected to the habitat that the snapper themselves occupy. The standard nursery hypothesis mechanisms of protection from predation or increased food abundance [2], may therefore not apply. Alternatively, the value of habitat structure to juvenile snapper may potentially be related to energetic shelter from water flow that habitat structure could provide to snapper, thereby allowing them to access higher fluxes of their pelagic prey in tidally driven water flow [15].

In the present study we further explore this hypothesis by making detailed observations of how post–settlement snapper associate with habitat structure and the water flow environment around this structure. We utilised Artificial Seagrass Units (ASUs) to standardise the structure available to post–settlement snapper and used a multi–camera set up to describe how post–settlement snapper spatially distributed themselves around this structure and the water flow features it created. Fine scaled water flow measurements were also taken from around the ASUs to understand the potential energetic refuging benefits associated with different locations that post–settlement snapper utilised.

## Materials and methods

**Fig 1:**
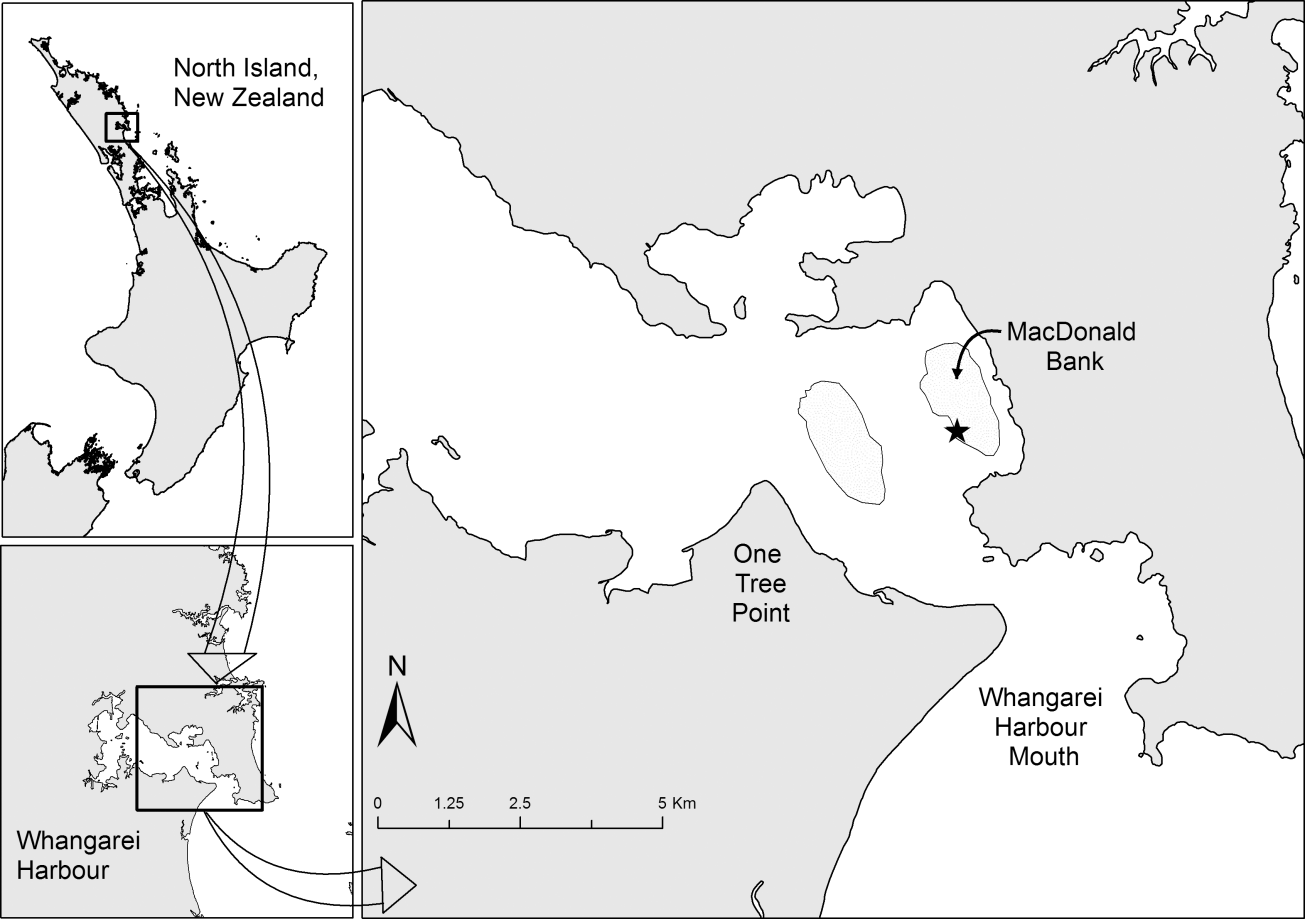
Map of Whangarei Harbour. Intertidal sand banks indicated by light grey shading and the location of the Artificial Seagrass Unit array denoted by a black star. North Island, New Zealand and Whangarei Harbour inset.

Experiments were conducted within Whangarei Harbour, northeastern New Zealand (Fig 1). Whangarei Harbour, is a mesotidal estuary with a semi-diurnal tide, and has neap and spring ranges of 1.3 m and 2.8 m, respectively. The harbour contains many shallow subtidal sand banks and flats suitable for conducting experiments on juvenile fishes, but we selected a site at MacDonald Bank (174.491° E, 35.810° S) for the present study for several reasons. Specifically, MacDonald Bank offers an extensive area of sand flats of appropriate depth to conduct experiments, it is devoid of natural structure and artificial habitat placed on MacDonald Bank attracts high densities of post–settlement snapper, it has reasonable water clarity for making observations, and can only be accessed by boat, reducing the potential for interference. Furthermore, MacDonald Bank experiences an average water flow velocity of c. 20 cm s^-1^, typical of the tidally dominated flows expected in the estuarine areas where post-settlement are abundant [11], and also typical of where post-settlement snapper are abundant within Whangarei Harbour [15, 17]. In December 2015 (the time of peak snapper spawning [11]) we deployed 10 1.8 m × 1.8 m ASUs at a water depth of c. 0.4 m at low tide. ASUs were separated by 20 m. Previous investigations have demonstrated that post-settlement snapper are attracted to ASUs in high densities, and are resident to patches separated by c. 10 m [12], so we treated ASUs as independent replicates. Earlier versions of the ASUs that we have constructed utilised artificial plants with wire stems. Because the primary purpose of the present experiment was to assess the response of snapper to water flow that may be modified by habitat structure, we altered ASU construction to include ribbon, which more naturally mimiced seagrass blades by being pushed down towards the sea bed with increasing water flow. Each ASU that we constructed in the present study incorporated a plastic mesh grid with bunches of ribbon (c. 30 cm long by 1 cm wide) tied to the mesh grid. All ASUs had a shoot density of 1820 blades m^-2^, corresponding to the upper range of subtidal seagrass densities in northeastern New Zealand [6]. ASUs were secured in place on the sand flats with metal pegs. We attempted to orient each ASU so that two of its edges ran parallel with the direction of the flooding and ebbing tide. Tidal current direction, however, does vary throughout the tidal cycle, and furthermore, may not be 180 degrees opposite for ebbing and flooding tides. We therefore orientated ASUs to minimise this error. No collections of animals or plants were made as part of this research, forgoing the need for a field permit or animal ethics approval.

### Video camera deployments

**Fig 2:**
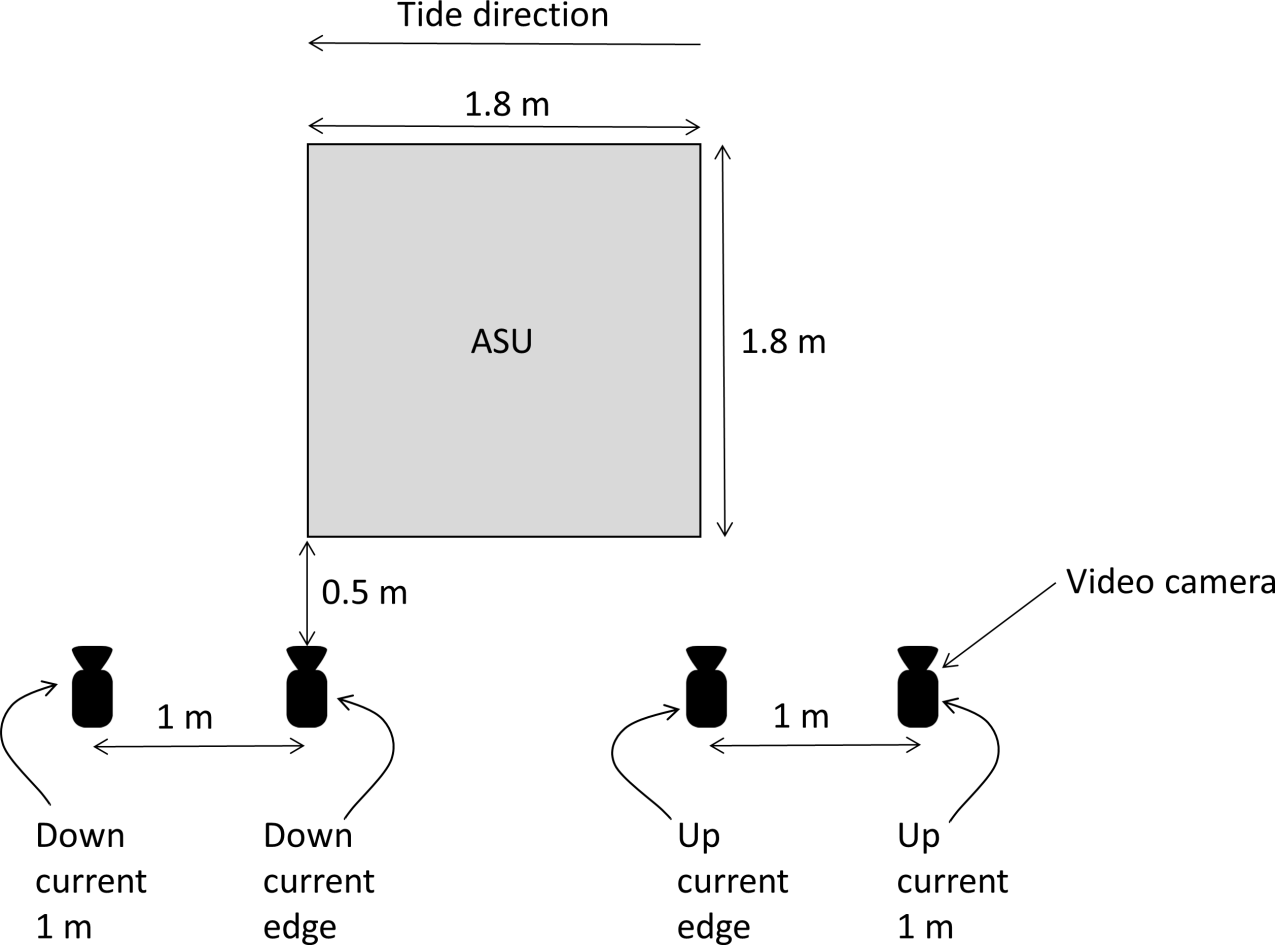
Aerial view layout of video cameras positions used to film post–settlement snapper associated with an ASU. Camera position naming indicated for just one water flow direction. Camera naming reverses on the opposing tidal direction. No camera deployments encompassed a change in tidal direction.

To understand how post–settlement juvenile snapper spatially orient to patches of habitat structure and the water flow field around that habitat we filmed juvenile snapper associated with ASUs using GoPro Hero 3 cameras. One camera deployment was conducted on each of the 10 ASUs during March 2016, the time when the abundance of post–settlement snapper in shallow nursery habitats should be at its peak [11]. For each camera deployment four cameras filmed an ASU (see Fig 2 for camera position layout) providing simultaneous footage (and information on post–settlement snapper abundance/behaviour) for four different sections around the ASU. All four cameras were positioned (held in place using 15 cm high steel frames) in the same plane relative to the ASU, 50 cm from its edge. All cameras were pointed perpendicular to that edge of the ASU. Camera deployments (and associated footage) generally lasted three to four hours, and did not overlap with low or high tide (i.e. water flow was always in the same direction within a deployment). This camera setup therefore provided observations of post–settlement snapper around each ASU in four different positions relative to the direction of water flow (hereafter termed: down–current 1 m, down–current edge, up–current edge, up–current 1 m).

When each camera began recording, and before it was deployed in place adjacent to an ASU, we initially filmed a wristwatch. This not only provided a reference for the time of day for video footage, but also allowed us to synchronise video footage from each of the four cameras that were simultaneously filming an ASU. We used a subsampling procedure to analyse video footage. This provided discrete time periods within which response variables could be categorised, and reduced the total amount of video footage that needed to be analysed. We followed a procedure of viewing one minute of footage and then skipping nine minutes. Because we had synchronised the video footage, one minute time periods represented the same actual periods of time across the four cameras. While viewing video footage we recorded three types of responses: (1) the maximum number of post–settlement snapper observed within the one minute time period. While we were not able to determine the exact length of snapper present in the video footage, the seagrass blades could be used for scale. During March there should be a c. 6 cm or c. 100% difference in the length of post-settlement and 1+ snapper [18], providing adequate resolution to categorise snapper into age classes. While snapper larger than the post–settlement stage (i.e. 1+ or greater) were observed around the ASUs on the video footage, they were not the focus of this investigation as they are not dependent on these nursery habitats. (2) The number of post–settlement snapper that were in the water column (here defined as not within 30 cm of the seabed or artificial seagrass). (3) Any predation attempts on post–settlement snapper. Only post–settlement snapper at a focal distance of c. 70 cm or less (i.e. between the camera and the fringe around the closest edge of the ASU) were counted. Water visibility was estimated for all one minute observation periods, and counts were not used if visibility dropped below 70 cm. For the cameras positioned in line with the edges of the ASU, their fields of view did not overlap with each other, but did overlap with the two cameras placed one metre further out. Therefore, we only counted post–settlement snapper in the outer half of the field of view for the cameras placed one metre from the ASU edge. These counts were then doubled to make them comparable with the counts made for the cameras placed in line with the ASU edge.

#### Video camera deployment statistical analysis

Because we had repeated observations from three to four hour video deployments at each ASU, we needed to account for the potential of temporal autocorrelation. Therefore, for each video camera position we performed separate autocorrelation analyses for post–settlement snapper abundance, and the number of post–settlement snapper within the water column. These analyses suggested that autocorrelation was acceptable (less than a 95% confidence interval centred around a value of no autocorrelation) when data was lagged by a multiple of four. We therefore thinned our data by systematically retaining every fifth record.

We initially explored data for a relationship between the two primary response variables (abundance of post–settlement snapper and the proportion of post–settlement snapper observed within the water column) and water velocity (as measured by the onsite acoustic Doppler current profiler (ADCP)) using 2^nd^ order polynomial quantile regression splines fitted through the 50^th^ percentile of the data [19]. Formal analysis consisted of generalised linear models (GLMs), with camera position (four levels, as detailed in Fig 2) and tide direction (flood or ebb) both treated as fixed factors, with ASU as the replicate. For post–settlement snapper abundance a quasi–Poisson error distribution was used, whereas for the proportion of post–settlement snapper within the water column a binomial error distribution was used. Plots of residuals from the final models were used to assess the assumptions of normality and homoscedasticity. Where statistically significant differences were detected, individual differences were identified by adjusting p–values using Tukey’s honest significant difference post hoc tests.

### Water flow measurements

Water flow was measured around the ASUs by two different deployments of equipment. During March 2016 we deployed a Nortek 2MHz Aqudopp (ADCP). This provided water velocity information that specifically related to our video observations of post–settlement snapper recorded during the same period. The ADCP was positioned near the middle of the line of ASUs, but about 50 m to their seaward side. From the ADCP we used water velocity measurements at 30 cm above the seabed (about the same elevation as the post–settlement snapper) in 10 minute time bins. We then matched these water velocity measurements to the one minute observation periods used to analyse the video footage.

The second deployment of equipment to measure water flow was conducted over a two day period during June 2016. The purpose of this deployment was to characterise the influence of the ASU on the hydrodynamic conditions that post–settlement snapper would experience around ASUs. To achieve this high-frequency velocity measurements were made using Acoustic Doppler Velocimeters (ADVs). To resolve the vertical flow structure, velocity measurements were collected at 15, 23, 26 and 38 centimetres above the seabed (cmab). The measurements from the 4 elevations form a vertical profile (hereafter referred to as a profile) of the three-dimensional velocity field. Velocity measurements at each elevation were collected at 25 Hz in four minute bursts, with an interval between bursts of five minutes. During the off period the height of the ADV was manually adjusted by a diver. Overall a total of 14 profiles were collected.

**Fig 3:**
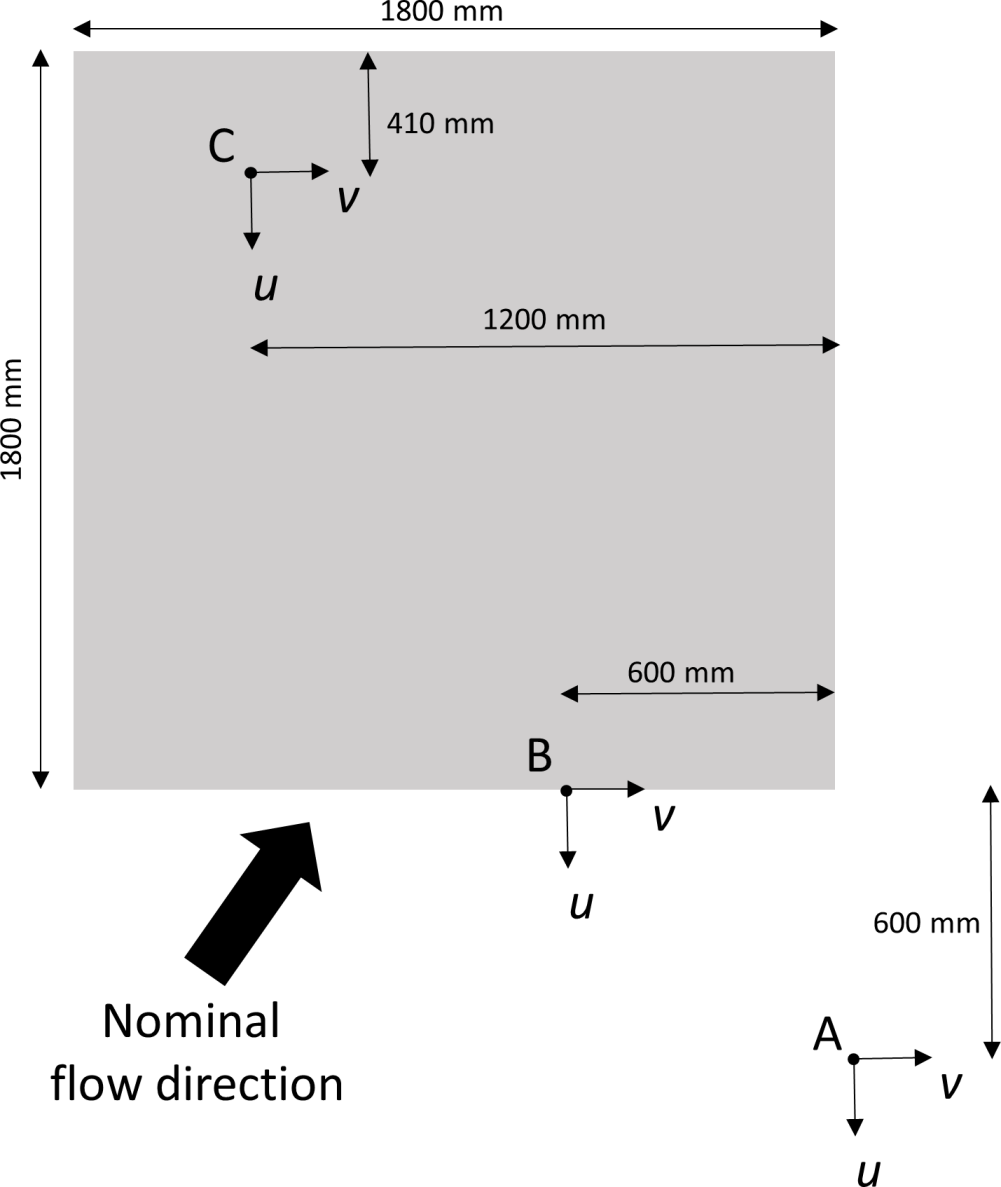
**Schematic diagram of experimental setup for the ADV measurements showing the position of three measurement locations (A, B and C) relative to the ASU.** Also shown is the orientation of the longitudinal (*u*) and transverse (*v*) velocity components relative to the orientation of the ASU. The vertical (*w*) velocity component was orientated in a positive direction away from the seabed.

Concurrent velocity profiles were collected at three locations, herein referred to as A, B and C (Fig 3). Location A was situated upstream of the ASU over a section of the bare seabed. Location B was located on the leading edge of the ASU, while C was positioned above the ASU at the trailing edge. These positions were selected to characterise the flow conditions before encountering, on encountering and at the back of the ASU.

Each ADV was attached to a stainless steel pole that was cantilevered to the main structure which ran parallel to the ASU. The main structure was securely fastened to the seabed via pipes secured into the seabed. To minimise disturbance each ADV was offset in the transverse direction to minimise flow disturbance by the upstream ADV, and flow measurements were only collected from one tidal phase (flood tide). The orientation of the horizontal (*u*) velocity component and the transverse (*v*) velocity component relative to the orientation of the ASU is depicted in Fig 3. The vertical (*w*) velocity component was orientated in a positive direction away from the seabed. In addition to the ADV measurements, full water column velocity profiles and wave statistics were measured using a Nortek 2MHz Aqudopp (ADCP) and an RBRconcerto (wave gauge), respectively.

### Analysis of water flow data

Following convention, each measured velocity component (*u*, *v* and *w*) was decomposed into a turbulent fluctuating component (denoted by a prime) and a time-averaged component (denoted by an overbar). Thus $u=\bar{u}+u^{\prime }$, $v=\bar{v}+v^{\prime }$ and $w=\bar{w}+w^{\prime }$. Here, the time average velocity of each component was defined as the low-pass filtered velocity component [20]. This is believed to be a better definition than the burst-averaged mean, as it removes any non-turbulent fluctuations that may contaminate the (turbulent) fluctuating signals.

To eliminate unwanted spikes in the ADV measurements the phase-space thresholding method [21] was adopted. The method was applied to each velocity component and iterated until the number of detected spikes was zero or the number of good points became constant. All detected spikes were replaced by the interpolation.

To minimise errors associated with sensor misalignment the “clean” (despiked) data were rotated into a streamline reference frame. The data were first rotated in the x–y plane to reduce the mean transverse velocity component to zero ($\bar{v}=0$) and then rotated in the x–z plane to reduce the mean vertical($\bar{w}=0$) velocity component to zero. Finally, a rotation in the x-z plane was perform to reduce $\bar{v^{\prime }w^{\prime }}$ to zero. The justification for these rotations was based on the assumption that well above the bed the flow is predominantly two-dimensional. Reflecting this, the three misalignment angles associated with the rotations were calculated for the upper two elevations in each profile. The rotation angles from each elevation were then averaged together and applied to all elevations within that profile.

The rate of turbulent energy dissipation (ε) was calculated using the inertial subrange method. At wavelengths sufficiently removed from both the large-scale eddies associated with turbulence production and the viscous-dissipation scale there exists a region known as the inertial subrange, in which turbulence is assumed to be isotropic on average. Without any energy sources or sinks in the inertial subrange, the flux of energy is equal to the rate of energy dissipation [22]. Using dimensional arguments and the isotropic assumption, horizontal and vertical turbulence energy spectra *E*_*u*_(*k*), *E*_*v*_(*k*), and *E*_*w*_(*k*) in the inertial subrange of wavenumbers take the form [23]:

$$E_{u}\left(k\right)=\frac{9}{55}\alpha_{1}\varepsilon^{2/3}k^{-5/3}$$ (1)

$$E_{v}\left(k\right)=\frac{9}{55}\alpha_{1}\varepsilon^{2/3}k^{-5/3}$$ (2)

$$E_{w}\left(k\right)=\frac{4}{3}E_{u_{1}u_{1}}\left(k\right)$$ (3)

where *α*_1_ = 1.5, *k* is the angular wavenumber, and *ε* is the rate of energy dissipation by viscosity. Eqs 1–3 are cast in terms of wavenumber. Typically, however, measurements of turbulence are made as a time series, which produces frequency spectra, not wavenumber spectra. Fortunately, Taylor’s “frozen-turbulence hypothesis” [24] provides a means to convert between frequency spectra and wavenumber spectra, with

$$k=\frac{2\pi f}{U}$$ (4)

and

$$E_{u,v,w}\left(k\right)=E_{u,v,w}\left(f\right)\frac{U}{2\pi }$$ (5)

where *f* is frequency in Hertz, *E*_*u*,*v*,*w*_ (*f*) is the frequency spectrum, and *U* is the eddy correlation velocity, which is assumed to be equal to $\bar{u}$.

Energy dissipation *ε* was calculated from measured time series by converting the frequency spectrum of the time series to a wavenumber spectrum using (4) and (5), and then fitting the right-hand side of (1), (2) or (3) (as the case may be) to the wavenumber spectrum to estimate *ε*. Due to the geometry of the ADV sensor head the vertical velocity component has a lower noise floor compared to the horizontal components, consequently *ε* was calculated using the fluctuating vertical velocity component.

## Results

### Video camera deployments

A total of 10 camera deployments were made (one on each ASU), with six deployments on flood tides, and four on an ebb tides. This provided > 183 hours of video footage (of which > 17 hours were observed), throughout which post–settlement snapper were generally abundant. Only one predation attempt was observed, where a kingfish (*Seriola lalandi*) aggressively pursued post–settlement snapper before quickly leaving the field of view. When this occurred post–settlement snapper quickly swam towards the ASU, with the majority of individuals hovering just above the canopy of the artificial seagrass blades. The number of post–settlement snapper around the ASU increased dramatically (from 5 to > 25 individuals) when this occurred, suggesting many snapper were outside of the field of view before the kingfish appeared. After the kingfish had left the post–settlement snapper quickly dispersed away from the ASU.

**Fig 4:**
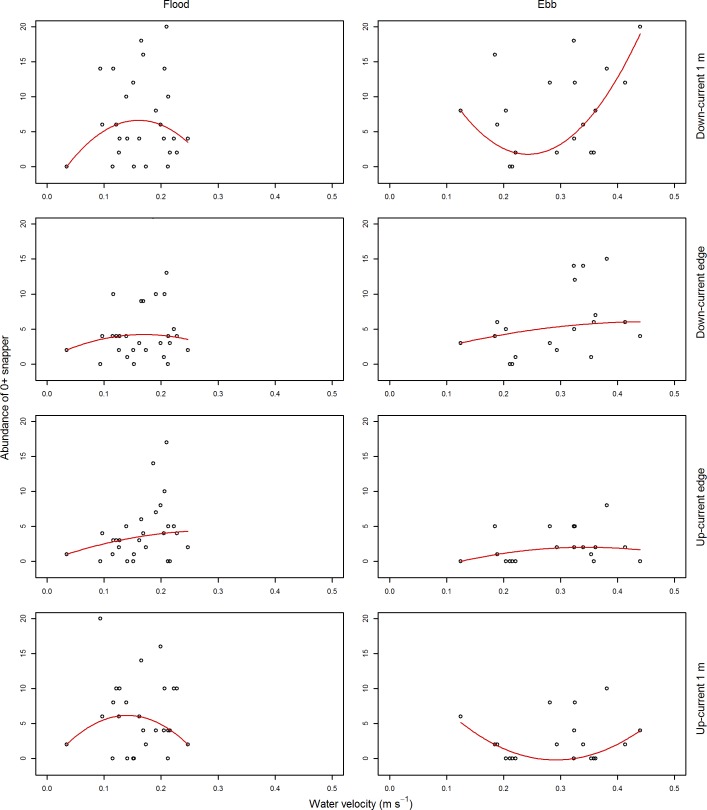
Abundance of 0+ snapper by water velocity. Snapper abundance is the maximum count observed within a one minute video sample. The left column of plots is for flood tide camera deployments, the right column for ebb tide camera deployments. Each row of plots represents snapper abundance as observed from a different camera position. Data presented have been subsampled to reduce temporal autocorrelation. The red line for each panel represents a 2^nd^ order polynomial quantile regression spline fitted through the 50^th^ percentile of the data.

**Fig 5:**
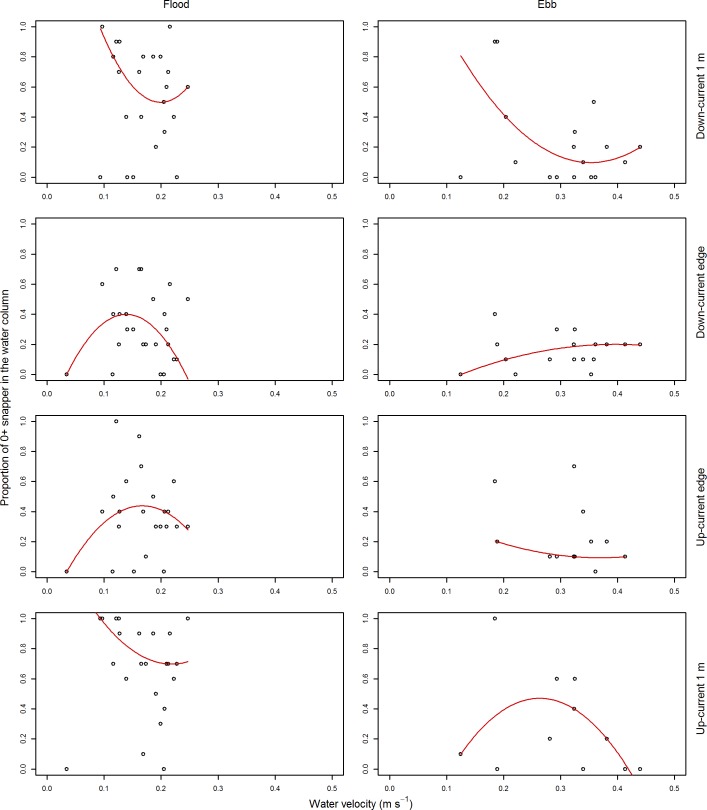
Proportion of 0+ snapper within the water column by water velocity. Proportion data are presented as open circles and were categorised for maximum counts from one minute video observations. The left column of plots are for flood tide camera deployments, the right column for ebb tide camera deployments. Each row of plots represents the proportion of snapper in the water column as observed from a different camera position. Data presented have been subsampled to reduce temporal autocorrelation. The red line for each panel represents a 2^nd^ order polynomial quantile regression spline fitted through the 50^th^ percentile of the data.

The relationship of the two primary response variables with water velocity was weak; although there was some indication that the abundance of post–settlement snapper increased with water velocity (Figs 4 and 5). We therefore decided not to assess the influence of water velocity within our GLM models, due to the weak relationship with our response variables described above, and the low replication that would be associated with water velocity had it been included within the GLMs (around five data points to assess the relationship with water velocities at each ASU).

**Fig 6:**
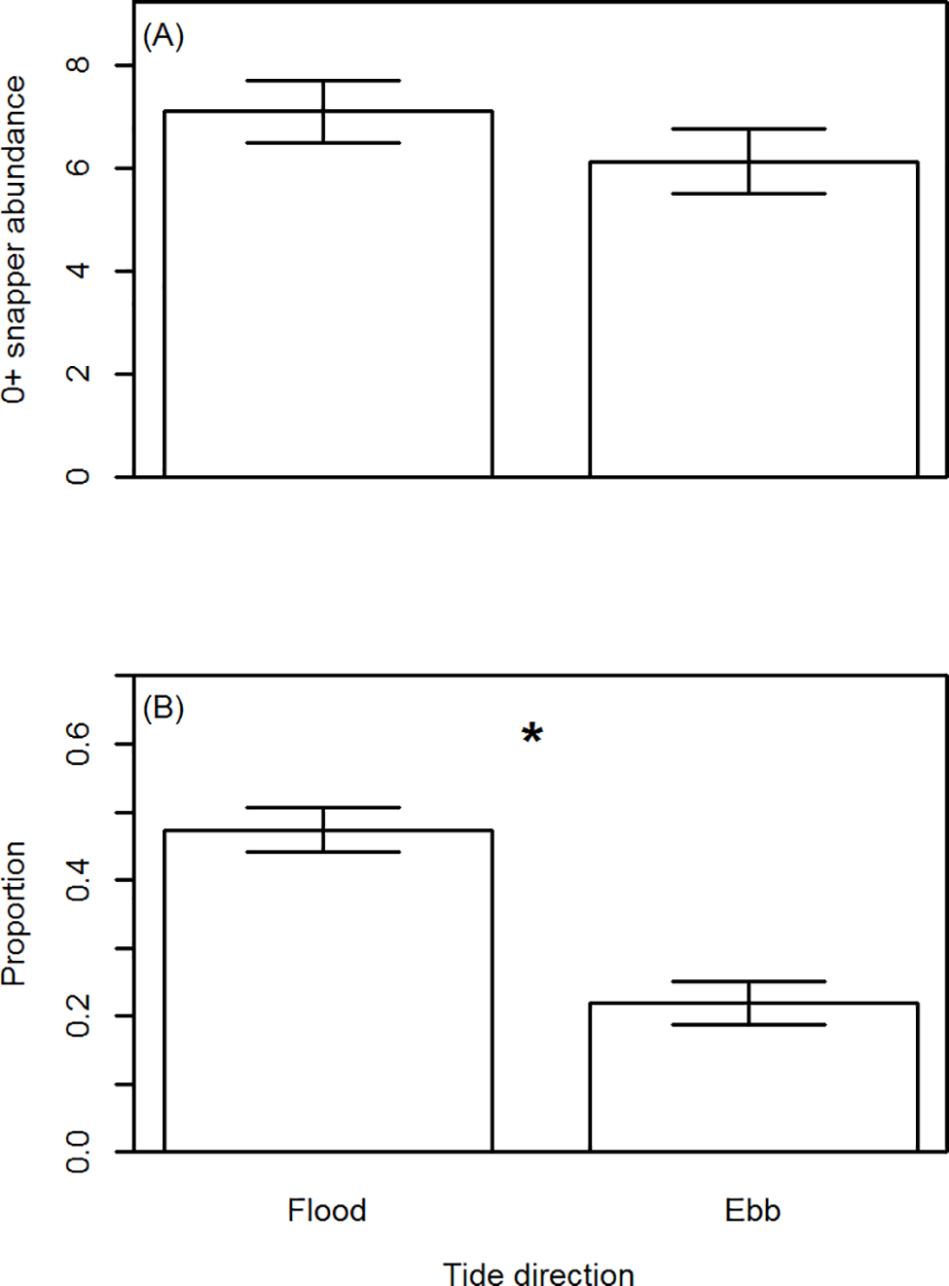
**Abundance of 0+ snapper (A), and the average proportion of 0+ snapper within the water column by tide direction (B).** Abundance data are the average of maximum counts observed from one minute video segments. Error bars are ±1 standard error. * denotes a significant difference (GLM analysis).

**Fig 7:**
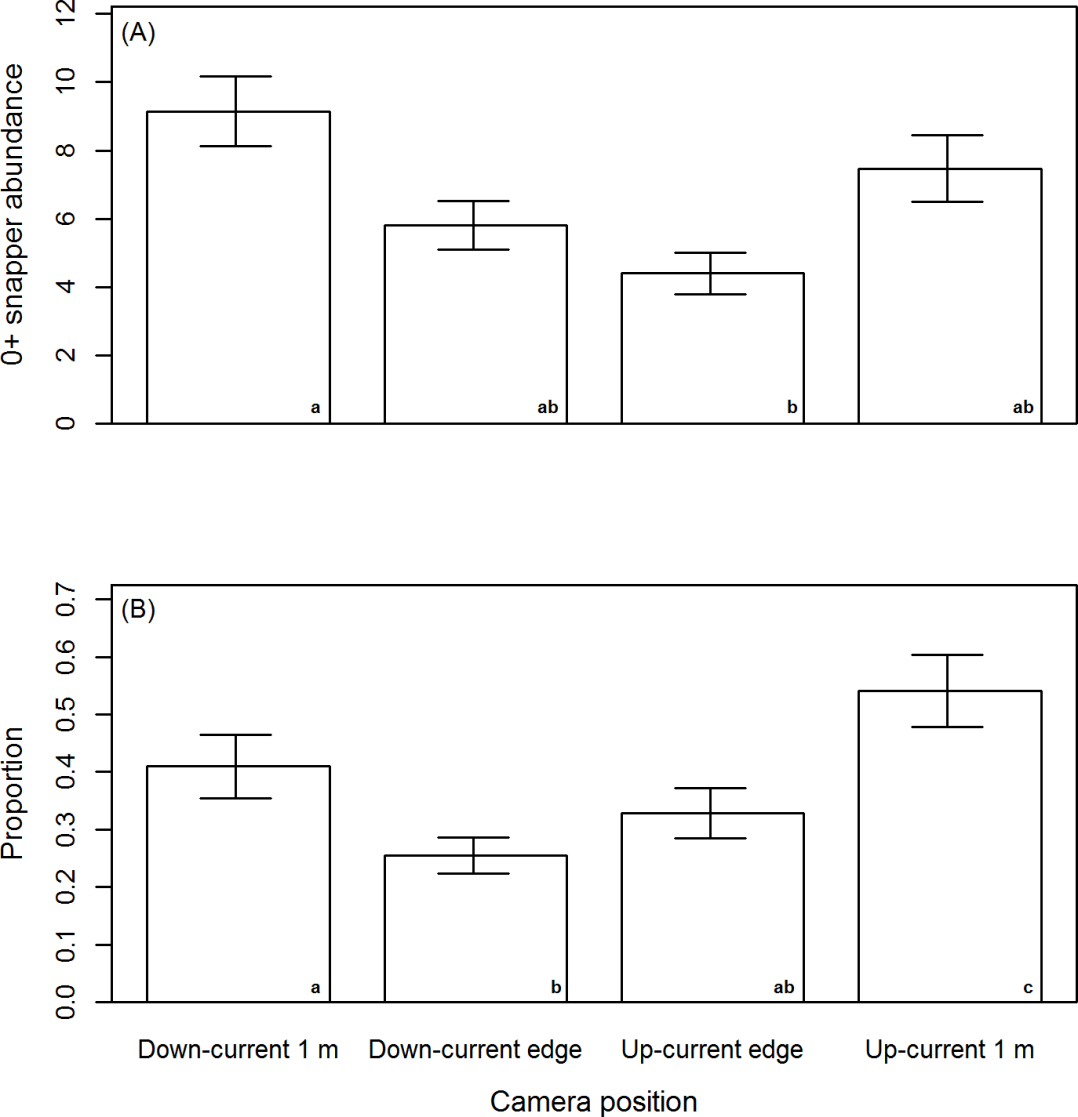
**Abundance of 0+ snapper (A), and the average proportion of 0+ snapper (B) within the water column by camera position.** Abundance data are the average of maximum counts observed from one minute video segments. Error bars are ±1 standard error. Camera positions with matching lowercase letters denote no significant difference upon multiple comparison.

The abundance of post–settlement snapper was non–significantly higher on flood vs. ebb tides (Fig 6A, GLM: *df* = 1, *F* = 3.57, *p* > 0.06), and significantly differed with camera position (Fig 7A, GLM: *df* = 3, *F* = 4.32, *p* < 0.02). This camera position effect was due to significantly higher abundance of post–settlement snapper at the down–current 1 m camera compared to the up–current edge camera (Tukey’s multiple comparison) (Fig 7A). There were no significant interaction effects between tide direction and camera position (*p* > 0.30).

The proportion of post–settlement snapper observed within the water column was significantly higher on flood vs. ebb tides (Fig 6B, GLM: *df* = 1, χ^2^ = 64.24, *p* < 0.00001), and significantly differed with camera position (Fig 7B, GLM: *df* = 3, χ^2^ = 65.95, *p* < 0.00001). This camera position effect was due to significantly higher proportions of post–settlement snapper within the water column at the up–current 1 m camera compared to all other cameras, and at the Down–current 1 m camera compared to the down–current edge camera (Tukey’s multiple comparison) (Fig 7B). There were no significant interaction effects between tide direction and camera position (*p* > 0.17).

### Water flow measurements

**Fig 8:**
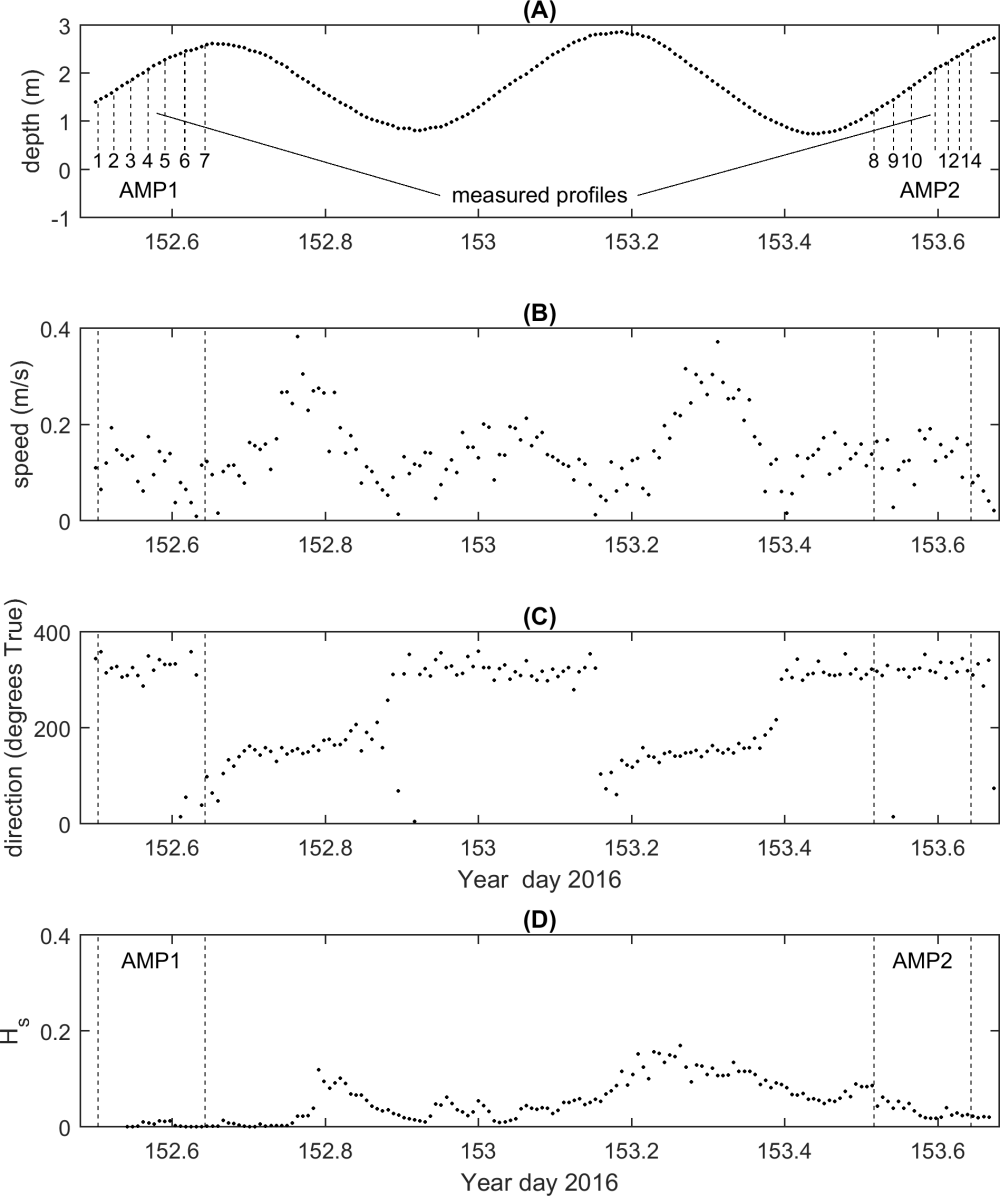
ADCP measurements of the background conditions experienced during the ADV deployments. Panels: (A) water depth, (B) current speed, (C) current direction (in oceanographic convention “flowing to”) and (D) significant wave height (*H*_*s*_). The timing of when the water flow profiles were collected is indicated on panel (A).

Background current conditions measured by the ADCP during the two day June 2016 deployment demonstrated that our field site had a water depth of between a few tens of centimetres at low tide to around 2.8 m at high tide (Fig 8A). Tidal currents at the study site were ebb dominated. Peak current speeds during ebb tides were typically < 50 cm s^-1^, whereas during the flood tide, current speeds were typically < 30 cm s^-1^ (Fig 8B). Current speeds during ADV measurement period 2 (AMP2) were slightly higher than those experienced during ADV measurement period 1 (AMP1), with a consistent current direction occurring across both of the ADV measurement periods (Fig 8C). AMP1 took place during a calm period of no waves, whereas AMP2 took place towards the end of a period of increased wave action (Fig 8D). However, significant wave heights (*H*_*s*_) measured during AMP2 were less than 5 cm.

**Fig 9:**
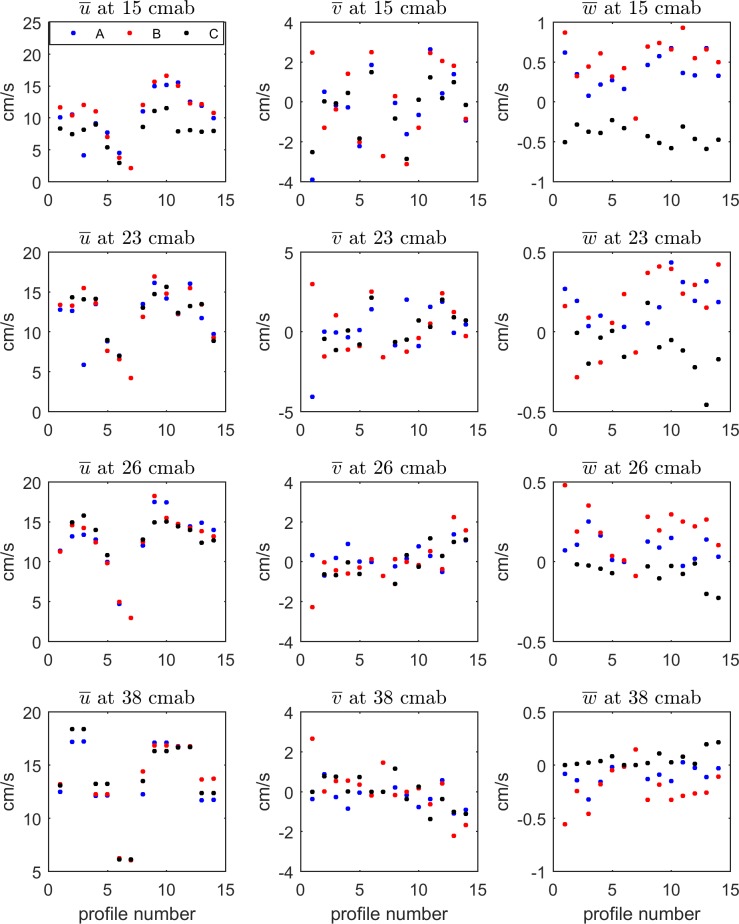
Vertical distributions of mean water velocity around an ASU. $\bar{u}$ is longitudinal velocity, $\bar{v}$ is transverse velocity, and $\bar{w}$ is vertical velocity, all measured at locations A, B and C. Profile number refers to the time period when each set of water velocity measurements were collected (AMP1 (profiles 1–7) and AMP2 (profiles 8–14), but see Fig 8 for a graphical representation). Measurements were made with ADVs during a two day deployment in June 2016.

A total of 14 water flow profiles were collected (profiles 1 to 7 and 8 to 14 were collected during AMP1 and AMP2 respectively) (Fig 9). At the lowest measurement height (15 cmab, which is about the height of the edge of the seagrass canopy when it is depressed by water flow) the measurements demonstrated a consistent and significant reduction in the mean longitudinal velocity ($\bar{u}$) at the back edge of the ASU (Location C) compared to the locations outside and on the leading edge of the ASU (locations A and B, respectively). Higher above the bed, profiles of $\bar{u}$ converged and there was no consistent difference between the three measurement locations. At the upper measurement elevations the mean transverse velocity ($\bar{v}$) and mean vertical velocity ($\bar{w}$) were approximately zero, which is consistent with the assumption that high above the bed the flow is predominantly two-dimensional. There were no consistent differences in the vertical profiles of $\bar{v}$ measured at the three locations. As expected, measurements of the vertical velocity component ($\bar{w}$) were much smaller than the horizontal velocity components. At 15 cmab there were small differences in $\bar{w}$ between the three locations. On encountering the leading edge (B) there was an increase in flow away from the bed (relative to locations A and B), while at location C the negative value of $\bar{w}$ indicated a flow in the opposite directions towards the bed. However, the differences in $\bar{w}$ were small and could have resulted from sensor misalignment, rather than any real differences in flow speeds across the ASU.

**Fig 10:**
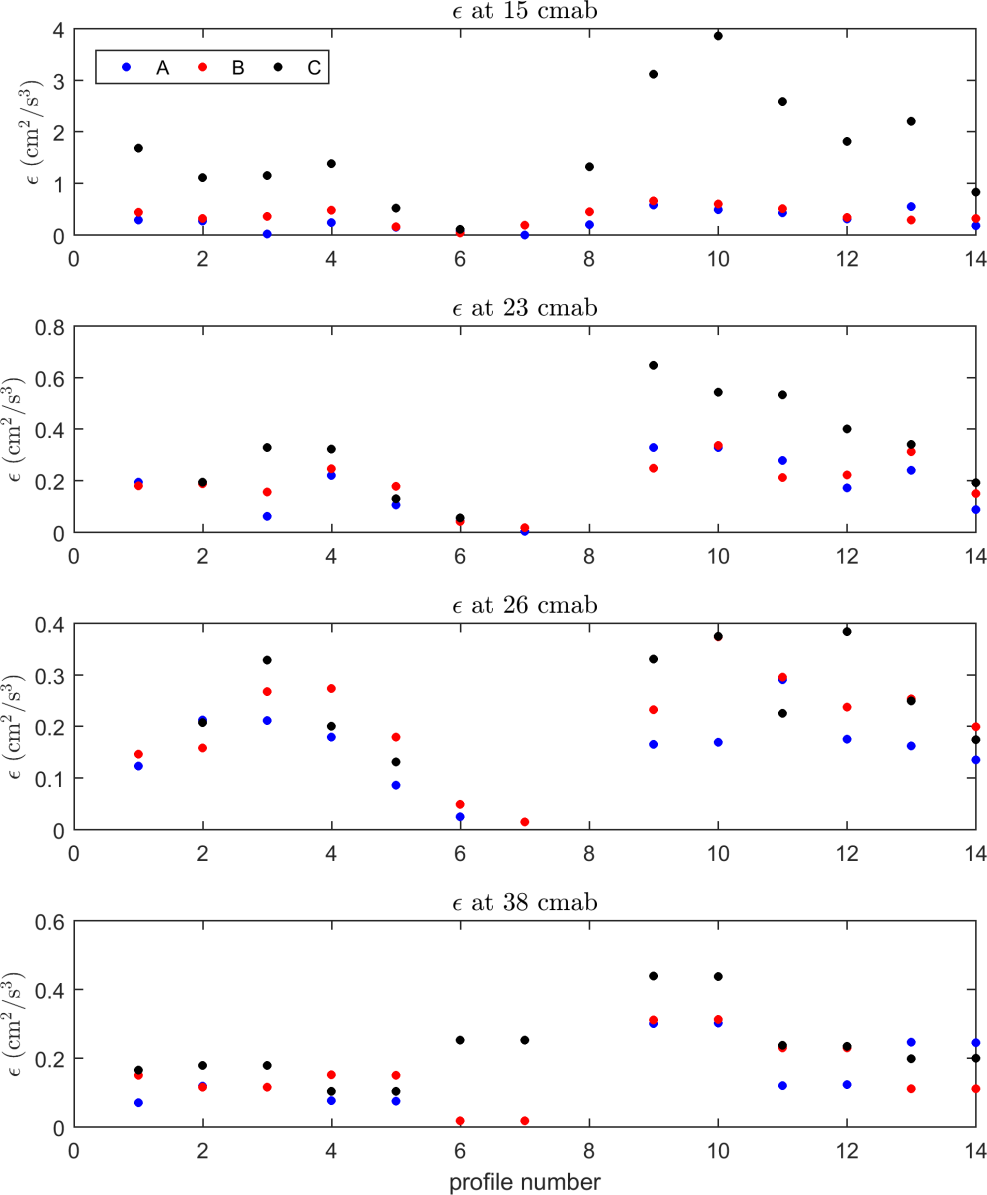
**Vertical distributions of turbulence energy dissipation (ε) measured at locations A, B and C.** Profile number refers to the time period when each set of turbulence measurements were collected (AMP1 (profiles 1–7) and AMP2 (profiles 8–14), but see Fig 8 for a graphical representation). Measurements were made with ADVs during a two day deployment in June 2016.

Turbulence energy dissipation (ε) rates at 15 and 23 cmab were consistently greater at the back edge of the ASU (location C) compared to those observed outside (A) and on the leading edge of the ASU (B) (Fig 10). There was also some indication that at 15 cmab dissipation was greater at the leading edge compared to outside of the ASU. At higher elevations it was difficult to discern consistent patterns between measurement locations. As expected dissipation decayed with elevation.

## Discussion

Understanding how nursery habitats function to support juvenile fish is an important part of conserving, restoring, or managing these habitats to sustain fish population productivity [9, 10]. For post–settlement snapper, we know that their abundance is strongly and positively related to the presence of habitat structure [12–14], and our previous research suggested that this relationship may be determined by a balance between access to planktonic food items and energetic shelter from water flow [15]. What the present study demonstrates is that there was spatial pattern in how post–settlement snapper related to the water flow environment around structure, suggesting they may be responding to refuge from water flow. Snapper were, however, also abundant at distances of more than a metre away from the ASUs that we deployed, and many snapper were up in the water column where they wouldn’t receive any refuge from water flow.

In terms of water flow, our results are consistent with other studies that have investigated how hydrodynamics are affected by permeable canopies such as seagrass. With regard to mean flow, previous studies [25, 26] have shown that flow speeds within the canopy are reduced relative to an unvegetated-upstream position, whereas above the canopy flow speeds are enhanced. At our lowest measurement height (15 cmab, which is about the height of the edge of the seagrass canopy) the measurements demonstrated a consistent and significant reduction in the mean longitudinal velocity ($\bar{u}$) at the back edge of the ASU compared to the locations outside and on the leading edge of the ASU. This result is consistent with the results from previous studies as discussed above. Our results, however, did not show a consistent increase in flow speeds above the canopy. Previous studies [26, 27] have also shown that strong velocity shear and significant increased turbulence intensity occur in the vicinity of the canopy-water interface. This increase in turbulence intensity is consistent with our measurements, which have shown that the turbulence energy dissipation (ε) rates at 15 and 23 cmab were consistently greater over the ASU compared to those observed outside and on the leading edge of the ASU.

The description of water flow above suggests that ASUs modified water flow in a way that could offer an energetic refuge for post–settlement snapper. Specifically, if snapper positioned themselves at the back of the ASU (and most likely further down–current as well) and close to the level of the seagrass canopy, they would experience the benefits of reduced flow speed. In addition, turbulence is also dissipating at a faster rate at these locations, which may also be beneficial to fish. Our video footage suggested that snapper may be responding to these water flow features. The highest abundance of snapper occurred a metre down–current of the ASU, with the lowest abundance on the up–current edge. Other fish species are also known to shelter behind structures in such a way to refuge from water flow [28–30]. For snapper to receive a flow refuge, however, they would also need to position themselves at a low elevation. While c. 60% of snapper down–current of the ASU were within 30 cm of the seabed, and may have been receiving some water flow refuge, that also implies that c. 40% were not receiving any flow refuge benefit.

In contrast with abundance, the proportion of post–settlement snapper within the water column was highest a metre up–current of the ASU. It may seem counter intuitive that snapper would predominantly position themselves away from the seabed in the location where any water flow refuging benefits would occur right next to the seabed. Flow refuging alone, however, may not be driving this relationship. If snapper were in the water column to access the drift of planktonic food items, being up–current of conspecifics associated with the same habitat patch may provide a higher food intake rate, as suggested for other fish species [31, 32]. We also observed a difference in the proportion of snapper within the water column a metre down–current of the ASU compared to the down–current edge, but it is not immediately clear what may be driving this difference. Aside from location relative to the ASU, the proportion of snapper within the water column was also higher on flood compared to ebb tides. If zooplankton abundance is replenished by flood tides (i.e. filter feeders within estuaries can reduce the abundance of plankton imported on flood tides [33–37]), then being within the water column during flood tides may be an effective feeding strategy.

Although post–settlement snapper are habitat–dependent [12–14], the present study demonstrates that at a fine spatial scale they are only loosely related to the water flow environment created by structure. While some snapper do position themselves close to the structure and will receive an energetic refuge advantage, other factors are also likely to be important. For example, a proportion of snapper positioned themselves within the water column, especially on the up–current side of the structure and on flood tides, indicating that pelagic feeding may also be determine how snapper position themselves. It is possible that post-settlement snapper alternate their position, feeding in the water column and up–current of structure, and sheltering close to the seabed and behind structure when they need to reduce energy expenditure. An alternative explanation, is that predation may be more important than we had originally thought (in a similar previous study we did not observe any predation events [15]). The one insight into predation from our video footage suggested that the ASU was of value to the post–settlement snapper because they swam directly and quickly towards it as the predator approached. While one interaction between post–settlement snapper and a predator is a rare event at the scale of video sampling that is possible, this does not mean that predator interactions are not significant at the scale that they are experienced by post–settlement snapper. In addition, the value of structure as a refuge from nocturnal predators has not been assessed, but could be important given the different suite of predators that will be active.

The identification and protection of the most productive nursery habitats is a key priority in managing fish populations, especially when resources are limited and have to be prioritised. While it is extremely challenging to quantify the contribution of different nursery habitats to the adult population [9, 10], understanding what makes a habitat or location valuable as a nursery may have utility. A mechanism–driven approach may reveal detail about the relationship between fish and the habitat they utilise, which could in turn provide a better understanding of what habitat features are likely to lead to higher habitat specific productivity. For example, in the present study we observed that post–settlement snapper positioned themselves both in the water column and up–current of structure as well as down–current of structure and near the sea bed (potentially alternating positions to maximise opportunity for pelagic feeding [15] and sheltering). Therefore, nursery habitat located in areas with appropriate water velocity are likely to provide a higher flux of food and support higher growth rates, which allow juvenile fish to advance through this vulnerable life stage [38]. Likewise, we also observed an instance where post–settlement snapper used structure to evade a predator, which may suggest that specific aspects of that structure which maximise predation avoidance might be beneficial [39]. Using the mechanisms that connect fish to nursery habitats may therefore be an efficient and ecologically informed approach to guiding management, but as the results of the present study suggest, it still has its own challenges.

## Supporting information

S1 File Data file containing water flow measurments and counts of post–settlement snapper around ASUs. (TXT)
